# Comprehensive proteome profiling of glioblastoma-derived extracellular vesicles identifies markers for more aggressive disease

**DOI:** 10.1007/s11060-016-2298-3

**Published:** 2016-10-21

**Authors:** Duthika M. Mallawaaratchy, Susannah Hallal, Ben Russell, Linda Ly, Saeideh Ebrahimkhani, Heng Wei, Richard I. Christopherson, Michael E. Buckland, Kimberley L. Kaufman

**Affiliations:** 1 Faculty of Science, School of Life and Environmental Sciences, The University of Sydney, Sydney, NSW 2006 Australia; 2 Discipline of Pathology, Sydney Medical School, The University of Sydney, Sydney, NSW 2006 Australia; 3 Brain and Mind Centre, The University of Sydney, Camperdown, NSW 2050 Australia; 4 Department of Neuropathology, Royal Prince Alfred Hospital, Camperdown, NSW 2050 Australia

**Keywords:** Extracellular vesicle, Glioblastoma, Proteomics, Invadopodia, Exosome, Annexin a1

## Abstract

Extracellular vesicles (EVs) play key roles in glioblastoma (GBM) biology and represent novel sources of biomarkers that are detectable in the peripheral circulation. Despite this notionally non-invasive approach to assess GBM tumours in situ, a comprehensive GBM EV protein signature has not been described. Here, EVs secreted by six GBM cell lines were isolated and analysed by quantitative high-resolution mass spectrometry. Overall, 844 proteins were identified in the GBM EV proteome, of which 145 proteins were common to EVs secreted by all cell lines examined; included in the curated EV compendium (Vesiclepedia_559; http://microvesicles.org). Levels of 14 EV proteins significantly correlated with cell invasion (invadopodia production; r^2^ > 0.5, p < 0.05), including several proteins that interact with molecules responsible for regulating invadopodia formation. Invadopodia, actin-rich membrane protrusions with proteolytic activity, are associated with more aggressive disease and are sites of EV release. Gene levels corresponding to invasion-related EV proteins showed that five genes (annexin A1, actin-related protein 3, integrin-β1, insulin-like growth factor 2 receptor and programmed cell death 6-interacting protein) were significantly higher in GBM tumours compared to normal brain in silico, with common functions relating to actin polymerisation and endosomal sorting. We also show that Cavitron Ultrasonic Surgical Aspirator (CUSA) washings are a novel source of brain tumour-derived EVs, demonstrated by particle tracking analysis, TEM and proteome profiling. Quantitative proteomics corroborated the high levels of proposed invasion-related proteins in EVs enriched from a GBM compared to low-grade astrocytoma tumour. Large-scale clinical follow-up of putative biomarkers, particularly the proposed survival marker annexin A1, is warranted.

**Electronic supplementary material:**

The online version of this article (doi:10.1007/s11060-016-2298-3) contains supplementary material, which is available to authorized users.

## Introduction

The need for clinically useful biomarkers is becoming more apparent as the clinical management of glioblastoma (GBM) moves towards individualised therapy and adaptive trial designs. Extracellular vesicles (EVs) are stable, membrane-enclosed particles released from either the cell surface (microvesicles, 100–1000 nm) or from an endosomal route (exosomes, 40–100 nm). EVs are composed of an array of proteins, nucleic acids, lipids, and other metabolites that often reflect the cell of origin [1, 2], meaning they are excellent reservoirs of biomarkers. Importantly, GBM-derived EVs can cross the brain–blood-barrier and are detectable in the peripheral circulation. Profiling the composition of GBM-derived EVs may, therefore, offer a non-invasive means of assessing tumours in situ, e.g., to identify molecular signatures indicative of tumour progression, recurrence and treatment failure. A ‘liquid biopsy’ would be especially valuable for patients with primary brain tumours, where radiological findings can be ambiguous, i.e., pseudoprogression and neurosurgery carries a very real risk of complication.

Characterisations of cancer-derived EVs are gaining research momentum also to delineate the role of EVs in the tumour microenvironment. Interestingly, EVs offer an intercellular route to transfer oncogenic material that can change the genetic programme of non-malignant cells, with demonstrated functional consequences in transformed recipient cells related to proliferation, invasion, angiogenesis, chemoresistance and immune repression [3–7]. Studies have described extensive RNA expression analyses of glioma-derived EVs [3, 8, 9], however, proteomic profiles are currently limited. Reported protein studies have identified small numbers of proteins (2D-gel electrophoretic or antibody-directed strategies), however relevant to GBM biology, or analysed EVs from limited sources [4, 8, 10, 11]. From other cancer-derived EV studies, we know that EVs contain a subset of cellular proteins, some of which depend on the cell of origin while other proteins are EV-enriched.

We recently described a comprehensive GBM membrane proteome profile, including several invasion-related proteins that correlated with the cell’s ability to produce invadopodia (actin-rich cellular protrusions with proteolytic activity) under normal culture conditions [12]. Interestingly, invadopodia act as multivesicular endosome (MVE) docking sites and are a site exosome release, meaning the ability to form invadopodia could determine the release of exosomes [13]. Exosome secretion is an essential part of invadopodia biogenesis and maturation, including the release of key invadopodial metalloproteinase, MT1-MMP that degrades the extracellular matrix [13]. Inhibition of two major regulators of invadopodia formation decreased exosome release from squamous cell carcinoma cells [13]; in breast cancer, there are significant associations between cell invasion, invadopodia maturation and EV production [14]. Together these findings not only indicate that EVs are genuine invasion structures of cancer cells, but also point to the potential benefit of profiling EVs as an indirect way to dissect molecular mechanisms of invadopodia biogenesis and function in tumour invasion [15].

Here, we provide the most extensive GBM-derived EV protein profile, captured from six cell lines derived from GBM tumours using high-resolution mass spectrometry (MS). To identify candidate proteins associated with more aggressive disease, we performed correlation analyses between EV protein levels and the originating GBM cells’ ability to form invadopodia and then explored corresponding tumour gene expression levels in silico. The in vitro GBM EV proteome profile was then compared to glioma-derived EVs isolated from Cavitron Ultrasonic Surgical Aspirator (CUSA) fluid. The CUSA system is used to fragment and extract solid tumours from the central nervous system [16]. CUSA washings contain tumour tissue fragments that are routinely used in diagnostic pathology [17]; however, the fluid component of CUSA washings is typically discarded. Here we show that this surgical fluid represents a valuable and abundant source of brain tumour EVs. Comparative quantitative proteome analysis of EVs enriched from CUSA fluid collected during a high-grade (GBM) and a low-grade glioma surgical resection was also performed to substantiate the candidate invasion associated EV proteins identified in vitro.

## Results

### Characterisation of EVs derived from GBM cells in vitro

**Fig. 1:**
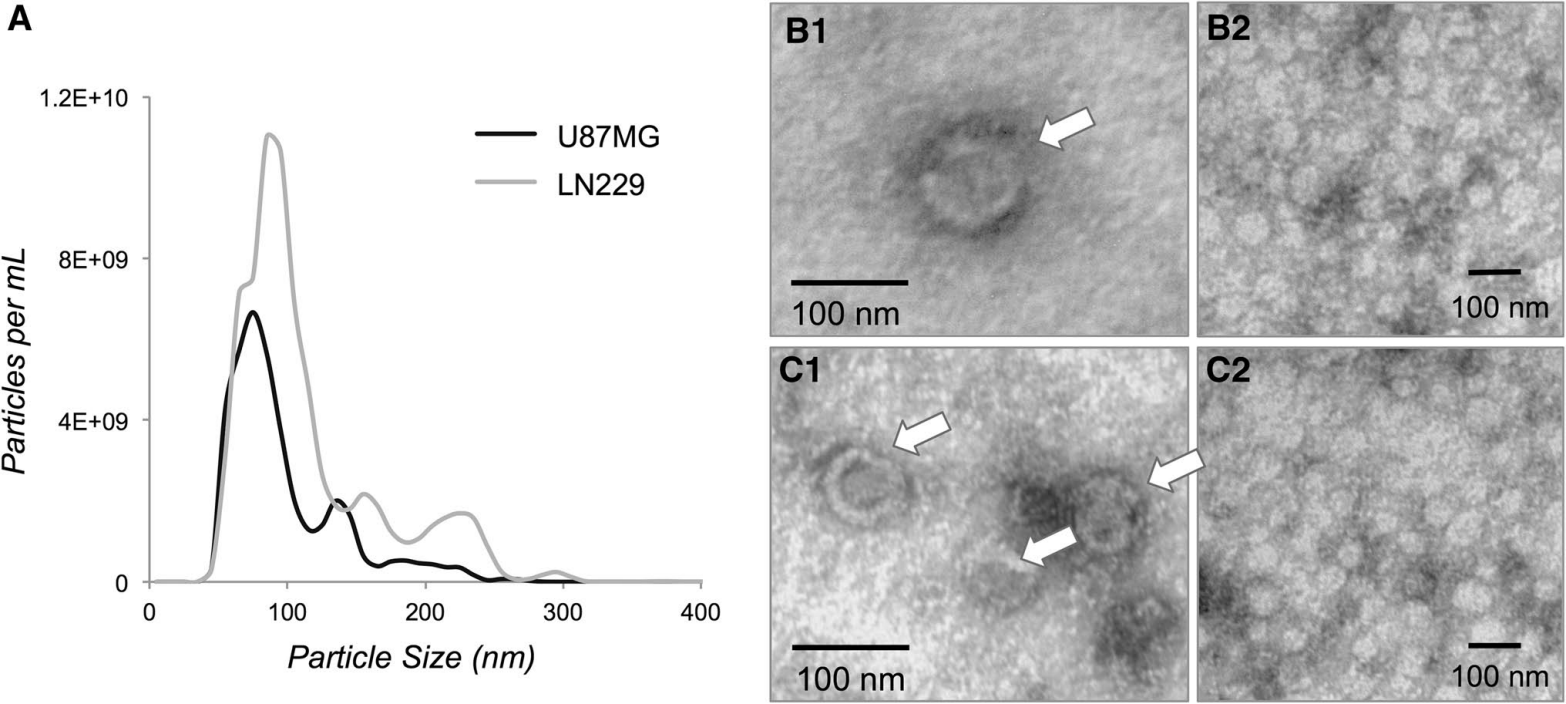
Characterization of GBM-derived EVs. **a** Size distribution of U87MG and LN229 EVs; *traces* represent triplicate experiments. Micrographs of (**b1, b2**) U87MG and (**c1, c2**) LN229 EV preparations show vesicles (indicated by *arrows*) with diameters of approximately 100 nm

**Fig. 2:**
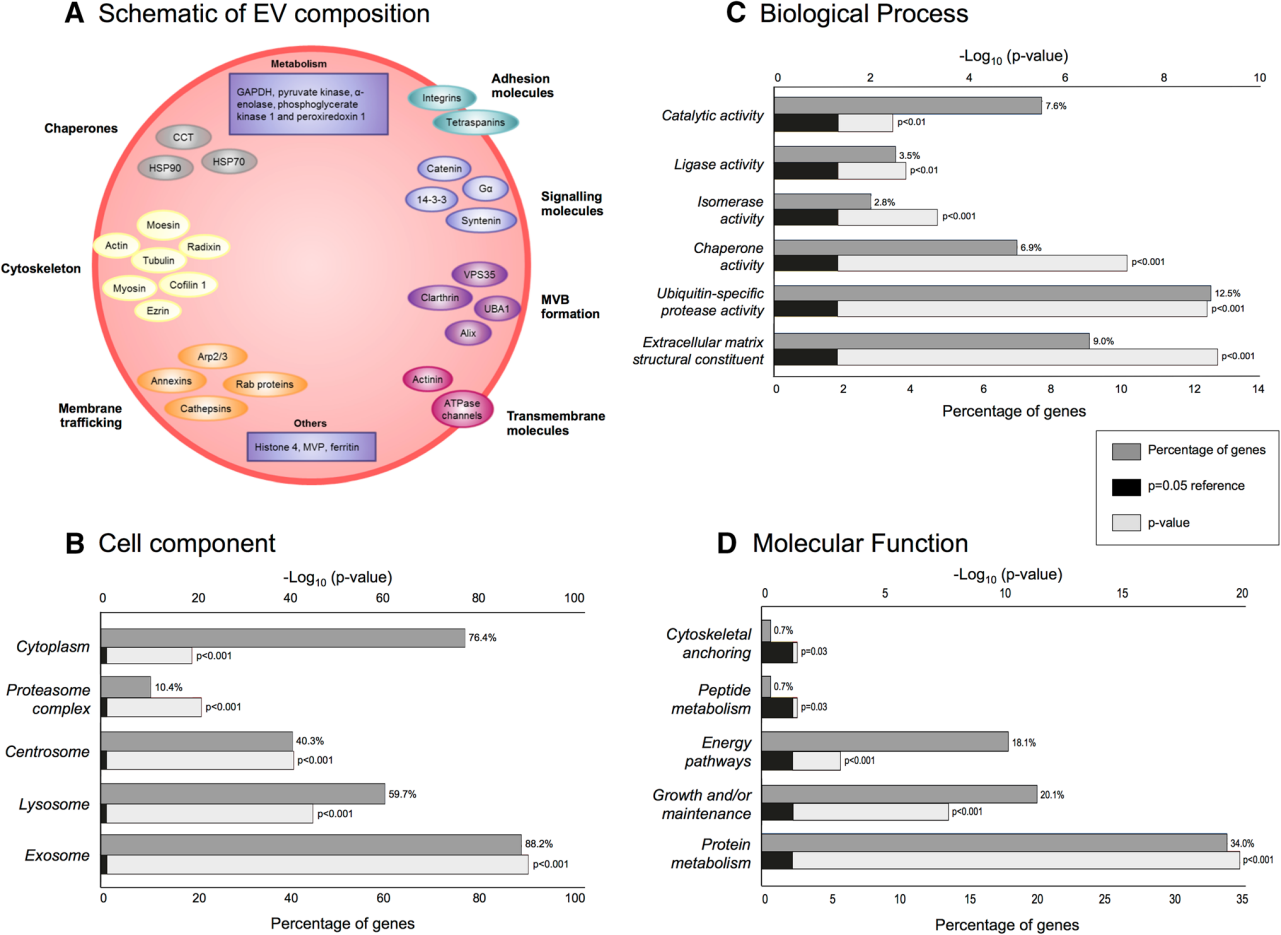
**a** Schematic of GBM-derived EV protein composition. Molecules are grouped based on their function or protein subclass determined by IPA. Identified EV proteins are involved in membrane trafficking and fusion processes including Ras-related protein 10 (Rab10), Rab7a, Rab5c, annexins A1, A2, A4, A5, A6, A11, cathepsins b and d (CTSB, CTSD), EH domain-containing protein 1, (EHD1), tripeptidyl-peptidase 2 (TPP2), and are markers for endosomes and lysosomes. Other protein groups include chaperones heat shock proteins (HSPA5, HSPA8, HSPA4, HSP90AB1, HSP90AA1, HSP90B1, HSPH1, HSPB1, HSPA1A, HSPA9), T-complex proteins (CCT2, CCT3, CCT4, TCP1, CCT7, CCT8, CCT5, CCT6A) and cytoskeletal proteins (α-actinin-1, α-actinin-4, myosin-9, α-tubulin-4a, actin and ezrin); cytosolic proteins are expected in EV profiles due to EV biogenesis and budding from the multivesicular body (MVB). Proteins involved in MVB formation, including exosomal marker, programmed cell death 6-interacting protein (PDCD6IP; ALIX) were also identified. Several transmembrane proteins were identified including integrins (β1, α3, αV) and CD44 as well as transporters, e.g., sodium/potassium-transporting ATPase subunit α1. Arp, actin related protein; MVP, major vault protein; Image adapted from [66]. **b**–**d** FunRich annotations based on 145 EV proteins common to all six GBM cells

The mean sizes of the U87MG and LN229 EV were estimated as 92.6 ± 1.2 and 109.9 ± 2.9 nm, respectively (Fig. 1a). Vesicles with diameters of approximately 100 nm were observed using TEM (Fig. 1b, c). Overall, 844 proteins were identified (≥2 peptides, 95 % confidence) of which 145 proteins (17.2 %) were common to EVs secreted by all cell lines (Supplementary Table 1; Vesiclepedia, dataset_599). We identified 15 of the top 20 previously reported exosomal proteins [18], eight of which were detected in all EV preparations.^1^ Cytochrome c, a marker for mitochondrial membrane contamination found in apoptotic blebs (i.e. much larger vesicles) [19], was not observed in any EVs. A schematic of the GBM EV proteome is provided (Fig. 2a) and describes a diverse set of proteins associated with MVBs (i.e., PDCD6IP and clathrin), cell adhesion, cytoskeleton, metabolism, membrane trafficking and chaperones. Primary sub-cellular localizations included significant enrichments of exosomal proteins (88.2 %; Fig. 2b). Identities of proteins novel to GBM EVs are annotated in Supplementary Table 1. This is the first account of osteonectin (SPARC; Vesiclepedia ID, VP_6678) and laminin subunit alpha-4 (LAMA4, VP_3910) proteins in cancer EVs, although corresponding mRNA species were documented in GBM EVs [3]. Gene names (145), corresponding to proteins common to all GBM EVs, were mapped in the IPA environment. Prominent up-stream regulators included NFE2L2 (p = 3.53E^−20^) and TP53 (p = 9.28E^−20^), with associations to 29 and 75 target molecules, respectively. Significant biological associations included cell growth/proliferation (81 molecules), cell fate (80) and cell-to-cell signalling (53); significant canonical pathways included the protein ubiquitination pathway (23/255), glycolysis I (7/25) and actin cytoskeleton signalling (11/217). Top scoring interaction networks showed functional association to cellular movement, cell fate, cellular growth and proliferation and cell-to-cell signalling (score 132, 78 molecules) and infectious disease, metabolic disease and amino acid metabolism (92, 61).

### EV proteins significantly correlated to GBM cell invasion

**Fig. 3:**
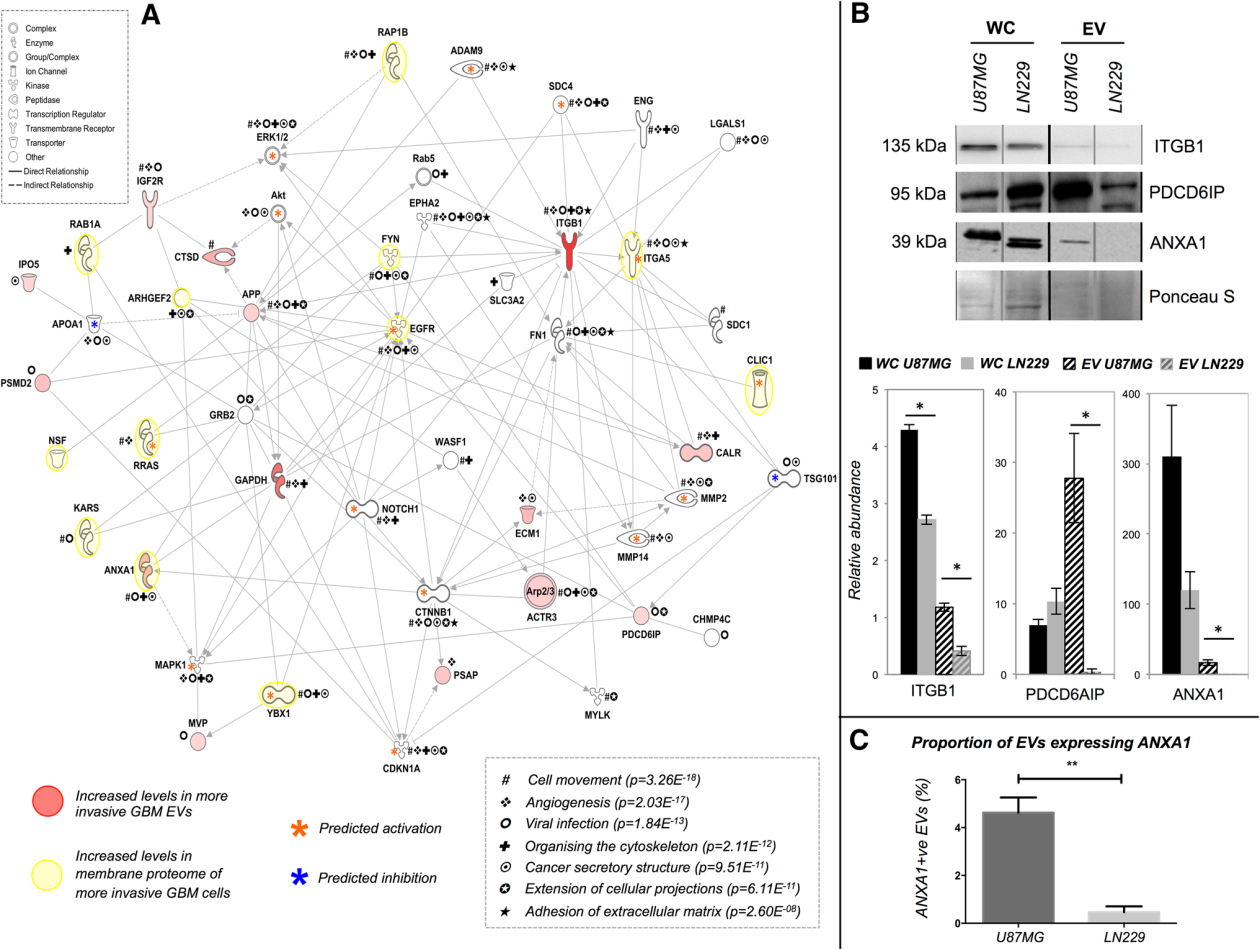
Interaction network EV proteins significantly correlated to GBM invasiveness. **a** Genes corresponding to 14 proteins were mapped in a network of 54 molecules using Ingenuity Pathway Analysis. Proteins with significantly higher levels in more invasive cells have red symbols. *Asterisks* highlight molecules associated with top scoring biological functions and canonical pathways, including tumour cell movement/invasion, cell-to-cell signalling, brain tumour/GBM signalling and formation and extension of cellular protrusions. **b** Confirmation of putative invasion-related EV protein changes. Whole cell (*WC*) and EV samples from the most (U87MG) and least (LN229) invasive cell lines were used to confirm significant abundance changes of ITGB1, PDCD6IP and ANXA1. Ponceau S blot stain was used as a loading control. *Bar charts* depict relative quantitation, where (*) indicates significance between the most and least invasive cells (p < 0.05) and *error bars* represent *standard error* of mean. **c** ANXA1 positive U87MG and LN229 EVs are shown as percentages of the total EV population, as measured by using a NanoSight CMOS camera and 532 nm laser in triplicate. Results represent the mean ± standard error of mean of three independent readings (**p < 0.01)

Abundance levels of 14 EV proteins significantly correlated to cell invasiveness (r^2^ > 0.5, p < 0.05, n ≥ 5; Table 1). Significantly associated diseases and cellular functions included cancer (14 molecules), neurological disease (8) cell-to-cell signalling and interaction (8) and cellular movement (8), with significant upstream regulation from *TP53, DYSF, PRL, CTNNB1* and *RAB7B* (6.33E^−08^ < p value <8.79E^− 06^). An interaction network was generated using the Path Explorer tool and included links to 12 membrane proteins^2^ previously detected at higher levels on more invasive GBM cells [12]; several genes corresponding to significant membrane proteins were also predicted to be activated in the generated network (Fig. 3a). Abundance changes of ANXA1, ITGB1 and PDCD6IP in EV lysates from the most (U87MG) and least invasive (LN229) cell lines were confirmed by Western blot (Fig. 3b). ITGB1 levels were also significantly higher in WC lysates of U87MG cells; an inverse relationship was observed for PDCD6IP. Higher ANXA1 levels were also indicated by increased fluorescence during nanosight particle tracking (Fig. 3c).

**Table 1:** Extracellular vesicle (EV) proteins correlate to the invasive potential of the originating GBM cell (r^2^ > 0.5; p < 0.05; n ≥ 5)

Acc.^a^	Gene	Protein name	r^2 b^	Unadjusted p-value ^c^	n^d^	Fold-change^e^
Q13200	PSMD2	26S proteasome non-ATPase regulatory subunit 2	0.85	0.0311	6	3.4
P61158	ACTR3	Actin-related protein 3	0.93	0.0201	5	2.6
P05067	APP	Amyloid beta A4 protein	0.82	0.0458	6	2.5
P04083	ANXA1	Annexin A1	0.89	0.0172	6	4.1
P27797	CALR	Calreticulin	0.82	0.0471	6	3.1
P07339	CTSD	Cathepsin d	0.90	0.0135	6	4.5
P11717	IGF2R	Insulin like growth factor receptor 2	0.92	0.0261	5	2.3
Q16610	ECM1	Extracellular matrix protein 1	0.93	0.0215	5	3.8
P04406	GAPDH	Glyceraldehyde-3-phosphate dehydrogenase	0.94	0.0059	6	7.7
O00410	IPO5	Importin-5	0.90	0.0394	5	2.3
P05556	ITGB1	Integrin beta-1	0.92	0.0255	5	11.2
Q14764	MVP	Major vault protein	0.88	0.0499	5	2.3
P07602	PSAP	Prosaposin	0.86	0.0297	6	3.1
Q8WUM4	PDCD6IP	Programmed cell death 6-interacting protein	0.90	0.0130	6	2.2

EV proteomes secreted by six GBM cell lines were quantified by averaging normalised precursor ion intensities. Invasive potentials were determined using the invadopodia assay [3]

^a^Accession numbers and gene names of proteins were retrieved from the Swiss-Prot database

^b^Pearson product momentum coefficient, r^2^, r^2^ > 0 indicates a positive relationship between invasiveness and protein abundance levels

^c^2-tailed significance threshold set to unadjust, p < 0.05

^d^ *n* number of cell lines where the protein was identified at 95 % confidence levels and ≥2 peptides

^e^Averaged precursor ion intensities from the most invasive divided by the least invasive cell line indicates extent of change

### Tumour transcript levels of putative invasion markers in independent glioma patient cohorts

**Fig. 4:**
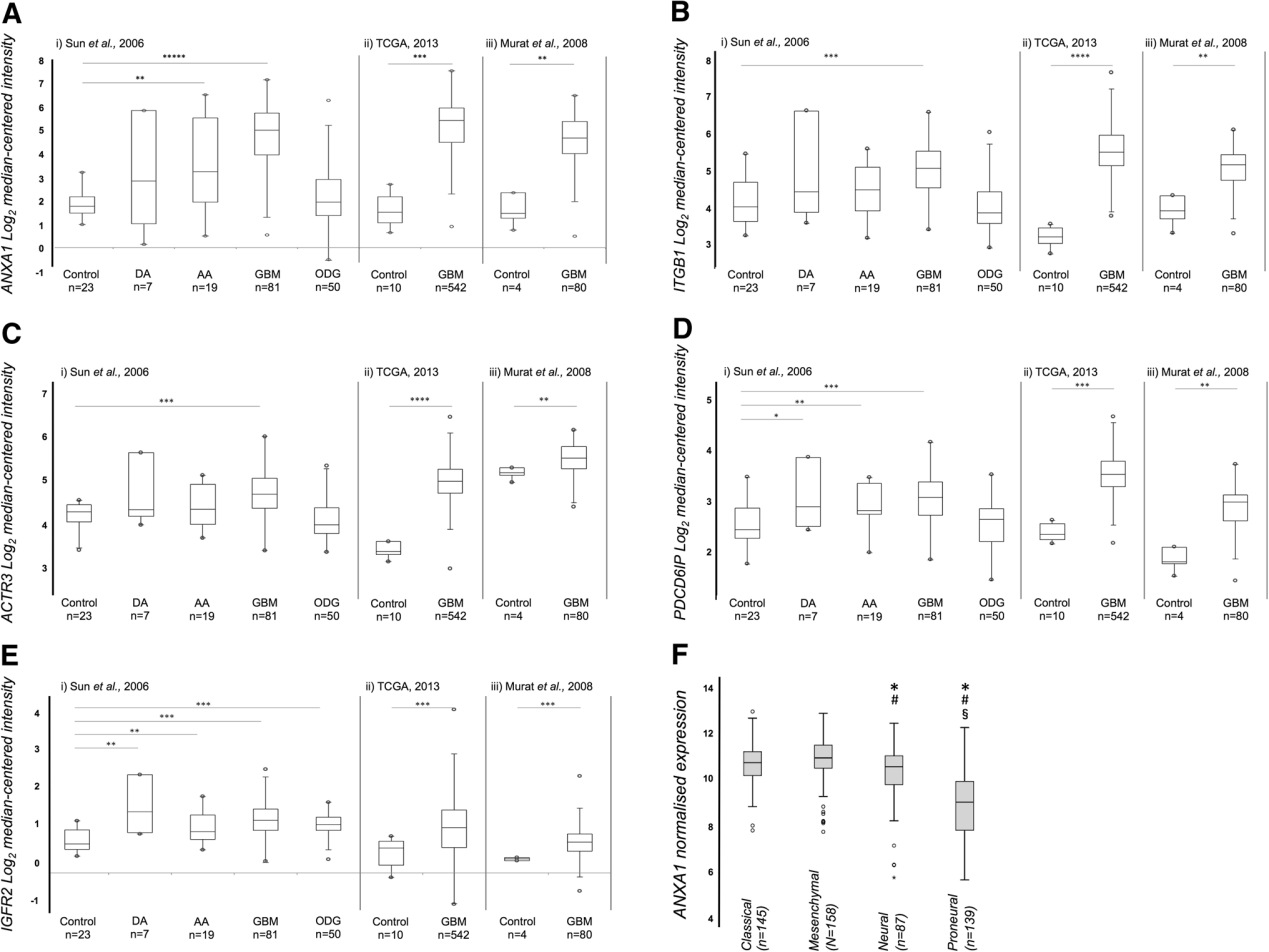
Tumour transcript levels of putative invasion markers in independent glioma patient cohorts. **a** *ANXA1*, **b** *ITGB1* **c** *ACTR3*, **d** *PDCD6IP* and **e** *IGFR2* levels [Human Genome U133 Plus 2.0 Arrays, cohorts *(i)* and *(iii)*; Human Genome U133A Array, cohort *(ii)*]. Expression levels generated by Oncomine are displayed as log_2_-median-centred ratio *box plots* comparing normal brain tissue to GBM or other less aggressive glioma tumours. Data from three cohorts *(i)* Sun et al. [67], *(ii)* TCGA [68] *(iii)* Murat et al. [69], refer to Supplementary Tables 3 and 4 for details; *n* is the number of samples, *open circles* represent maximum and minimum values; *error bars* represent 1.5× interquartile range; *p < 0.05; **p < 0.01; ***p < 1E^− 04^; ****p < 1E^− 11^. *ANXA1* levels were significantly higher in GBM compared with normal brain tissues across all three datasets, with 7.3-fold (p = 1.52E^− 26^), 11.7-fold (p = 2.50E^−09^) and 7.5-fold (p = 5.40E^−04^) increases in *(i), (ii)* and *(iii)*, respectively. *ANXA1* levels were also significantly higher in anaplastic astrocytomas (3.3-fold, p = 6.34E^− 04^), though to a lesser degree. *ITGB1* levels were significantly higher in GBM compared with normal brain tissues across all three datasets, with 1.7-fold (p = 3.94E^−07^), 4.4-fold (p = 5.0E^−12^) and 5.1-fold (p = 5.0E^−03^) increases in *(i), (ii)* and *(iii)*, respectively. *ACTR3* levels displayed the same trend, with higher expression levels in GBM across all three datasets, with 1.4-fold (p = 1.25E^−07^), 2.9-fold (p = 6.66E^− 13^) and 1.6-fold (p = 0.007) increases in *(i), (ii)* and *(iii)*, respectively. *PDCD6IP* mRNA levels were higher in GBM (1.4-fold, p = 2.25E^− 05^), diffuse astrocytoma (1.3-fold, p = 0.04), and anaplastic astrocytoma (1.3-fold, p = 0.009) compared with normal brain in dataset *(i)*. Compared to normal brain, GBM *PDCD6IP* mRNA was increased by 2.3-fold (p = 2.16E^−11^) in dataset *(ii)*, and 2.1-fold (p = 5.90E^−04^) in dataset *(iii)*. In dataset *(i), IGF2R* was significantly higher across four glioma subtypes compared to normal brain tissues, i.e., GBM (1.5-fold, p = 4.61E^−11^), diffuse astrocytoma (1.7-fold, p = 0.007), anaplastic astrocytoma (1.2-fold, p = 0.003), and oligodendroglioma (1.3-fold, p = 6.20E^−07^). In dataset *(ii), IGF2R* expression was higher in GBM compared to normal brain (1.6-fold increase p = 6.51E^−05^) and the same trend was observed in *(iii)* where *IGF2R* expression was 1.4-fold higher in GBM compared with normal brain tissue (p = 1.29E^−11^). f *Box plots* representing *ANXA1* normalised gene expression across the TCGA GBM classical, mesenchymal, neural and proneural transcriptional subtypes. *Open circles* represent maximum and minimum outlier values; error bars represent 1.5× interquartile range; (*) significant expression change relative to the classical subtype (*vs*. neural, p = 0.004; *vs*. proneural, p = 1.73E^− 27^); (^#^) significant relative to mesenchymal subtype (*vs*. neural, p = 6.76E^− 05^; *vs*. proneural, p = 2.02E^− 31^); (^§^) significant relative to the neural subtype (*vs*. proneural, p = 1.15E^− 12^)

Relative gene expression levels corresponding to 14 invasion-related EV proteins were analysed in silico to indicate whether these proteins might be clinically relevant. Transcript levels of *ANXA1, IGF2R, ITGB1, PDCD6IP* and *ACTR3* were significantly higher in GBM specimens, compared with normal brain across all three datasets (Fig. 4a–e). Significant increases in *IGF2R* were observed in diffuse and anaplastic astrocytomas and oligodendrogliomas, increased *PDCD6IP* in diffuse and anaplastic astrocytomas, and increased *ANXA1* in anaplastic astrocytomas relative to normal brain. Interestingly, significant differences in *ANXA1* were observed across the four TCGA GBM transcriptional subtypes, with significantly higher levels in classical and mesenchymal subtypes relative to neural and proneural tumours; proneural tumours displayed significantly lower gene levels compared to other subtypes (Fig. 4f).

### Cavitron Ultrasonic Surgical Aspirator (CUSA) fluid, a novel source of brain tumour EVs

**Fig. 5:**
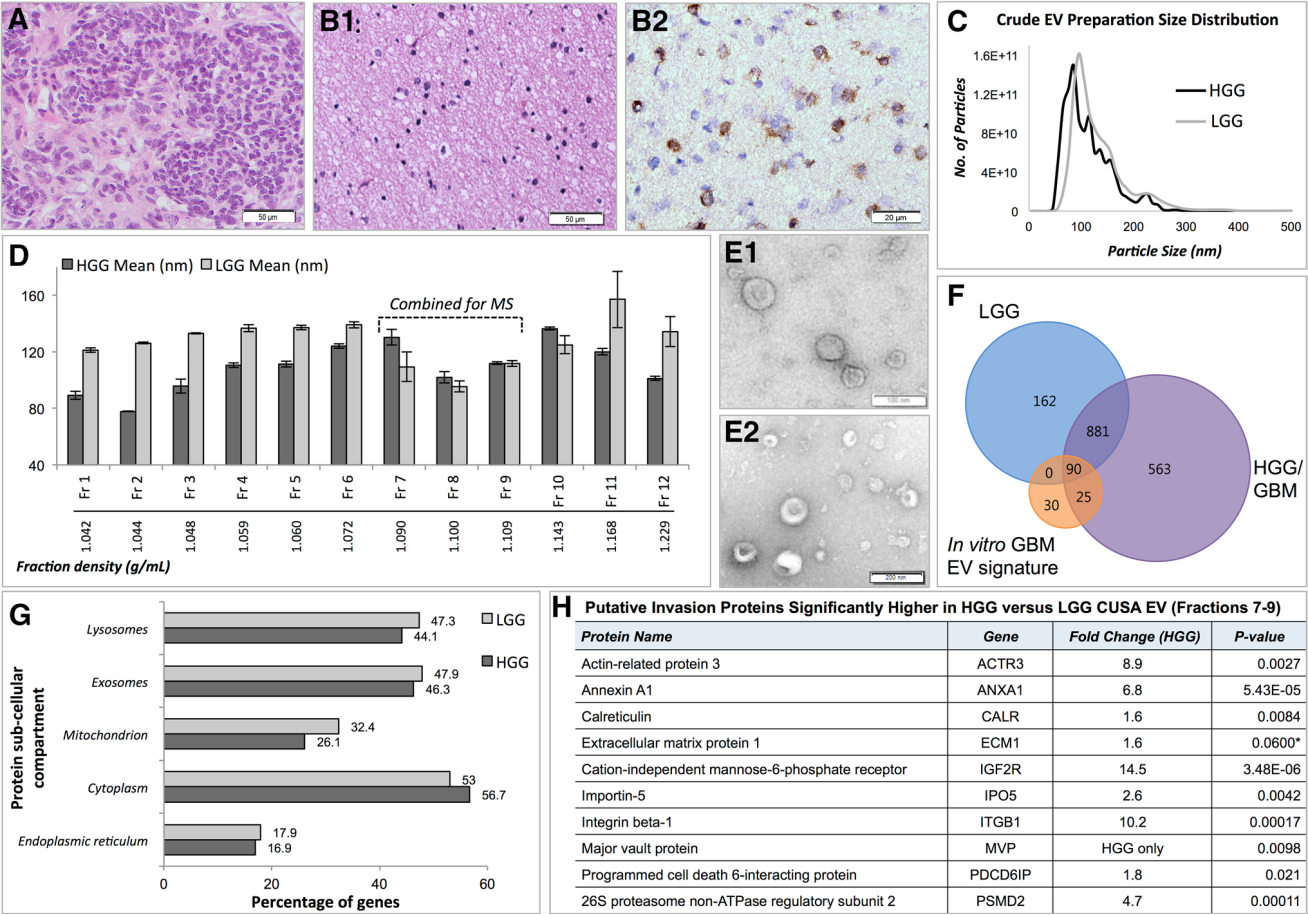
Cavitron Ultrasonic Surgical Aspirator (*CUSA*) fluid collected during High Grade Glioblastoma (*HGG*) and Low Grade Glioma (*LGG*) Surgical Resections. Haematoxylin and Eosin stained sections of tissue fragments recovered from CUSA washings collected during (**a**) HGG (WHO2007 Grade IV primary GBM) and (**b1**) LGG (WHO2007 Grade II diffuse astrocytoma) surgeries (*scale bar* 50 µm). **b2** The LGG tumour specimen was immuno-positive for *IDH1* (*R132H*) mutation (*scale bar* 20 µm). **c** Nanosight particle tracking analysis showed size distributions of particles in crude EV preparations from fluid recovered from HGG and LGG CUSA washings. **d** Mean sizes (nm) of particles isolated from Optiprep™ ultracentrifugation density fractions (Fractions 1–12), and corresponding densities (g/mL). *Error bars* indicate the standard error of mean. Transmission electron microscopy showed morphologies consistent with vesicles in combined density fractions 7–9 from (**e1**) HGG (*scale bar* 100 µm) and (**e2**) LGG (*scale bar* 200 µm). **f** Venn diagram depicts overlap of proteins identified at 95 % confidence levels by mass spectrometry (MS) in fractions 7–9 from HGG and LGG preparations, with the in vitro GBM EV signature proteins. **g** FunRich generated bar chart reveals percentage of genes corresponding to identified proteins in HGG and LGG fractions 7–9 corresponding to sub-cellular compartments. **h** Quantitative mass spectrometry analysis revealed nine putative ‘invasion’ proteins significantly higher in HGG compared to LGG CUSA-enriched EVs, and one protein with borderline significance (*). Fold changes are relative to the HGG sample

CUSA washings were collected during resections of a low-grade glioma (LGG) and a high-grade GBM (HGG) and tissue fragments processed for diagnostic histopathology (Fig. 5a, b). The HGG was confirmed as a primary WHO2007 Grade IV GBM tumour (*IDH1wildtype*) and the LGG, a WHO2007 Grade II diffuse astrocytoma (*IDH1mutant* immunopositive; Fig. 5b2). Crude EVs isolated from HGG (1.0 × 10^13^ particles/mL) and LGG (9.59 × 10^12^ particles/mL) CUSA fluid showed particle sizes with mean diameters of 107.9 ± 5.6 and 130.0 ± 1.8 nm, with large populations of 85 and 95 nm sized particles, respectively (Fig. 5c). As it was likely that the crude CUSA EV preparations contain tissue and cellular debris, including contaminating intracellular organelles, EVs were enriched further by density gradient ultracentrifugation and isolated from fractions with densities reflecting the reported range for EVs [20]. Fractions 7–9, corresponding to densities of 1.09–1.11 g/mL, contained particles with combined mean and mode diameters of 122.8 ± 5.6 and 95.2 ± 9.9 nm for HGG EVs and 134.3 ± 5.8 and 103.1 ± 8.7 nm for LGG EVs (Fig. 5d). TEM confirmed a vesicular morphology in the combined density fractions 7–9 from HGG (Fig. 5e1) and LGG (Fig. 5e2). Enriched EV fractions were then subject to quantitative MS analysis. We identified 1559 and 1133 proteins at 95 % confidence levels in at least two of three MS replicates in HGG and LGG EV (Fr7–9), respectively. Of these, 971 proteins were confidently identified in both samples (Fig. 5f). There was considerable overlap with the 145 in vitro GBM EV signature proteins, of which 115 were identified in HGG EVs and 90 in LGG EVs, including SPARC, however LAMA4 was not sequenced. Twenty-five EV proteins were identified in the HGG CUSA EVs alone and may be related to more advanced disease (indicated in Supplementary Table 1). While contamination of mitochondrial and endoplasmic reticulum proteins was observed, our analyses included the identities of 18 (LGG) and 19 (HGG) of the top 20 exosomal proteins (Supplementary Table 1), and approximately half of identified proteins had ‘exosomes’ as a sub-cellular compartment annotation (Fig. 5g). Of the 14 putative invasion proteins identified in the in vitro correlation analysis above, nine proteins (ANXA1, IGF2R, ITGB1, PDCD6IP, ACTR3, CALR, IPO5, MVP, PSMD2) were significantly higher in HGG compared to LGG CUSA enriched-EVs (p < 0.05; *Benjamin Hochberg adjusted p value significance threshold p* < *0.033*; Fig. 5h).

## Discussion

EV size distributions, morphologies and protein compositions indicate that exosomes are a predominant population in our preparations. Several identified proteins are involved in EV biosynthesis, including members of the ubiquitin-dependent complex ESCRT, i.e., vacuolar protein sorting-associated protein 35 and ubiquitin-like modifier-activating enzyme 1, suggesting that EVs analysed here originate from MVBs. Protein ubiquitination, the top scoring canonical pathway, is important for exosome formation especially during the recruitment of ESCRT machinery into MVB [21]. Although 0.2 µm filtration would theoretically remove microvesicles larger than 200 nm, their presence cannot be discounted for NTA measurements are less precise for larger vesicles (Fig. 1) and our in vitro EV preparations were not purified by density gradient ultracentrifugation.

To our knowledge, this is the first account of SPARC and LAMA4 proteins in EVs secreted by cancer cells, with previous observations restricted to normal saliva [22], bloods from healthy donors [23] or from patients with stable coronary artery disease [24], as well as cultured endothelial cells [25, 26] and embryonic stem cell-derived mesenchymal stem cells.^3^ TGBF1, also observed in EVs isolated from high-grade glioma patient sera [10], could be a candidate GBM EV marker. Despite the documented roles of TGFB1, SPARC and LAMA4 in GBM progression and invasion [27–29], only the SPARC protein was identified in the two clinical glioma EV preparations profiled here.

### Annexin A1, a potential EV biomarker predictive of GBM patient survival

We previously reported significantly higher ANXA1 protein levels in the membrane proteomes of more invasive GBM cells [12]. Increased *ANXA1* transcript levels were also observed in GBM and anaplastic astrocytoma tumours compared to normal tissue (Fig. 4) and high *ANXA1* expression identified a group of astrocytoma and GBM patients with reduced survival [12]. Interestingly, *ANXA1* expression levels change significantly across the TCGA transcriptional GBM subtypes with mesenchymal and classical tumours displaying the highest levels and proneural tumours (frequently *IDH1mutated*) the lowest. These differences may reflect the molecular disparities between the different tumour strata or perhaps simply, the reported differences in overall survival [30]. ANXA1 protein levels were also significantly higher in the HGG EVs compared with LGG EVs, which again may be associated with differences in tumour invasiveness between low- and high- grade tumours as well as the observed difference in *IDH1* mutational status (Fig. 5b2). Understanding the role of ANXA1 in GBM-derived EV is important in determining the influence of EV on the brain tumour microenvironment and role in tumour cell invasion. Further study of ANXA1 as a prognostic biomarker and anti-invasion target is warranted.

### ITGB1, part of the fibronectin receptor

Integrin β1 (ITGB1), a known EV protein, is important for invadopodia formation [31, 32] and *ITGB1* gene levels are elevated in GBM tumours (Fig. 4). ITGB1 has multiple direct and indirect interactions with other invasion-associated proteins, particularly ITGA5, which dimerizes with ITGB1 to form the fibronectin (FN1) receptor, α5β1 [33]. We recently showed that ITGA5 levels are significantly associated with GBM invasion and reduced patient survival [12]. Following FN1-mediated ubiquitination of ITGA5, α5β1 is sorted into MVEs via ESCRT machinery and destined for lysosomal degradation [34]. Although the orchestrated recycling of FN1-α5β1 (cycles of cell adhesion and detachment) is a requirement for migration, the fate of α5β1 is unknown. It is feasible that more motile cells endocytose more ITGB1, which is then sorted into MVEs that are shed as EVs. As more invasive cells express more ITGA5 on their surface [12] and secrete EVs with more ITGB1, the role of FN1-α5β1 in the GBM tumour microenvironment should be further delineated as it may offer an attractive therapeutic target.

### Increases in key invadopodia formation protein, ACTR3 and exosomal marker PDCD6IP

There was a significant association between high levels of PDCD6IP (also known as ALIX) and ACTR3 and increased invadopodia formation. The interaction network (Fig. 3) included links to several molecules with key regulatory roles in invadopodia formation, i.e., SRC, actin regulatory complex Arp2/3, WASF1 [35]. ACTR3 is an ATP-binding component of Arp2/3 and together with an activating nucleation-promoting factor such as Wiskott-Aldrich syndrome protein (WASP), WASF1 (WAVE) or WASH, it mediates actin polymerization and invadopodia formation [36]. PDCD6IP and ACTR3 are both involved in endosomal sorting, which is important for exosome biogenesis. As invadopodia are proposed as sites for exosome secretion [13], this could imply that more invasive or invadopodia-producing cells secrete more exosomes. This is supported by observations that tumours cells produce more exosomes per cell than normal cells [37] and *PDCD6IP* and *ACTR3* levels are higher in GBM tumours compared to normal tissue (Fig. 4).

### Intracellular Ca^2+^ regulation and exosome secretion

Calreticulin (CALR) levels were increased in EVs secreted by more invasive GBM cells. CALR is a critical regulator of Ca^2+^ homeostasis [38], its overexpression increases intracellular Ca^2+^ [39]. Increased cellular Ca^2+^ stimulates exosome secretion [40], which again supports the notion that more invasive GBM cells secrete more exosomes. Major vault protein (MVP) mRNA was previously observed in GBM-derived EVs [3] and significantly higher protein levels were identified in more invasive GBM EVs here. MVP facilitates the nuclear tumour-suppressing function of PTEN in a Ca^2+^ dependent manner [41]; nuclear PTEN is unable to inhibit PI3K signalling, leading to a more malignant phenotype [42]. Interestingly, EVs are highly enriched in vault RNAs [8] that complex with MVP to form the vault organelle that plays important roles in transport mechanisms, signalling and immune responses [42, 43]. MVP is upregulated during malignant transformation and tumour progression and has been linked to chemoresistance [42]. CALR was shown to promote invasion by increasing MMP-2 and MMP-9 [44] and is implicated in regulating radiosensitivity and radiation-induced apoptosis in GBM [45]. CALR is also a critical component of antigen processing and loading into MHC I [46]. Higher CALR levels in more invasive GBM EVs might be important for local and distant intercellular communication and have immunogenic modulatory effects.

CALR functions as a chaperone for amyloid beta A4 protein (APP) [47], also identified at significantly higher levels in EVs from more invasive cells. Increased APP protein levels were observed in GBM tumours [48] and APP metabolites are enriched in exosomes purified from brain tissues [49]. Increased APP was shown to up-regulate leucine-rich glioma inactivated-3 in rat astrocytes, which interacts with flotillin-1 to mediate APP trafficking, endocytosis and exosome formation in neuronal cells [50]. Elevated APP expression is also associated with gliosis and is the main component of the senile plaques; Alzheimer’s pathology is present in about half of all cases of GBM [51]. While APP seems to be a part of a poorly understood cell-contact signalling pathway [48], elevated APP levels in invasive GBM-derived EVs suggests that this communication occurs via EV delivery.

### Other EV invasion proteins

Insulin-like growth factor 2-receptor (IGF2R) was measured at higher levels in more invasive GBM EVs and tumour mRNA levels were higher in gliomas than normal brain (Fig. 4). Insulin-like growth factor-binding protein 2, a glioma marker linked to poor prognosis [52], binds to and modulates IGF2R. IGF2R also bind cathepsins [53] that are typically localised to lysosomes; EV biogenesis is now understood to involve pathways common to lysosome degradation [54]. Cathepsin D (CTSD) was also increased in EVs from more invasive cells, its release and activity is linked to glioma invasion [55], and may act directly by degrading local ECM structures or indirectly through activation of cysteine proteinases [56]. Interestingly, elevated CTSD serum levels correlate with glioma grade [57] and high *CTSD* transcript levels in GBM tumours is associated with reduced survival [58]. CTSD levels in circulating EVs might offer valuable, non-invasive prognostic information. ECM1 overexpression is associated with poor prognoses in breast, gastric and laryngeal cancer [59–62]. *ECM1* mRNA is enriched in GBM-EVs compared to cells [3]. Higher EV protein levels detected here as well as previous links to more aggressive cancer phenotypes suggest that ECM1 is an interesting target for further study.

While the phenotype of a cell or tissue correlates directly with protein expression, they may not correlate with mRNA levels [63, 64], therefore the expression levels of the nine invasion-associated proteins that did not show significance in silico may still be useful protein biomarkers. Along with the five proteins that did show concordance with mRNA levels in silico, CALR, IPO5, MVP and PSMD2 protein levels were significantly higher in HGG compared to LGG enriched EVs; ECM1 levels were also higher, however with borderline significance (p = 0.060).

### In vivo considerations for translational EV biomarker studies

Although the ability to detect appropriate biomarkers in the peripheral circulation is the sine qua non of a liquid biopsy, EVs isolated from peripheral blood pose two key problems during the initial discovery phase of biomarker development and should be considered for translation of the in vitro GBM EV protein signature described here. Firstly, the presence of high abundance proteins (albumin, immunoglobulins, transferrin and lipoproteins etc.) comprise ~99 % of the protein content of blood, masking the presence of low abundance proteins that are of major interest for biomarker discovery and make high throughput proteomic analysis of serum or plasma-derived EVs problematic [65]. Secondly, EVs are secreted by all bodily organs with a significant proportion in the blood being platelet-derived [9]. Tumour-derived EVs exist at relatively low concentrations within the blood compared to the total EV population [10]; high enough for targeted detection, but not sufficient for the bottom-up, high throughput analytical approaches for biomarker discovery [11]. This necessitates enrichment steps, which are still in the process of being standardised, precluding comprehensive and collaborative analysis in many cases [12]; the establishment of a ‘gold-standard’ for EV sourcing remains elusive [13]. An ideal EV source would need to deliver a relatively homogeneous mixture at high enough concentration that even low abundance particles could be detected and quantified. While the limitations of using immortalized cultured cells as models of complex heterogeneous disease are patent, there are no pan-GBM specific EV surface proteins described that would enable positive isolation methods from the blood. Thus, obtaining enriched sources of brain tumour EVs, i.e., from homogenized tumours, surgical aspirates or cerebrospinal fluid, presents the most plausible approach to translate the proposed GBM EV signature here as well as further test EV-associated biomarkers. Our preliminary analyses here indicate that CUSA fluid represents a valuable and abundant source of brain tumour EVs. Once confirmed, candidate biomarker proteins would also require further assessment to determine whether they are exploitable as biomarkers, i.e., that levels are above that of background soluble or normal EV levels in the peripheral circulation.

## Conclusions

A common set of 145 proteins was identified in EVs secreted by six GBM cell lines and may be useful for distinguishing GBM-specific EVs in the circulation. Many of the invasion-related EV proteins resolved are associated with key molecules involved in regulating invadopodia formation. Gene levels corresponding to five invasion-related EV proteins (*ANXA1, ACTR3, ITGB1, IGF2R* and *PDCD6IP*) were significantly higher in GBM lesions, with common functions relating to actin polymerisation and endosomal sorting. Several targets identified here warrant further testing as potential biomarkers, including the putative prognostic marker ANXA1. The role of FN1 and it’s integrin-α5β1 receptor in the GBM microenvironment should be further delineated and the inhibition of this association, e.g., by *volociximab* treatment, should be assessed. On the whole, these data indicate that more invasive GBM cells secrete more exosomes, a strategy that perhaps allows tumours to hijack their microenvironment and modulate anti-tumour immunity. Finally, we have identified CUSA washings as a novel source of brain tumour-derived EVs. The analysis of which could expedite the translation of clinically relevant blood-based biomarkers for GBM patient management.

## Electronic supplementary material

Below is the link to the electronic supplementary material.

Supplementary material 1 (DOCX 52 KB)

Supplementary material 2 (DOCX 210 KB)

Supplementary material 3 (XLSX 59 KB)

Supplementary material 4 (DOCX 48 KB)

Supplementary material 5 (DOCX 31 KB)

## Footnotes

1 Annotated in Supplementary Table 1.

2 ANXA1, ITGA5, EGFR, FYN, CLIC1, RRAS, ARHGEF2, RAB1A, RAP1B, YBX1, KARS, NSF.

3 Vesiclepedia dataset_126.
