# Supplementary material for: Comprehensive proteome profiling of glioblastoma-derived extracellular vesicles identifies markers for more aggressive disease
Source: J Neurooncol. 2016 Oct 21;131(2):233–44. doi: 10.1007/s11060-016-2298-3 (PMC5306193; doi:10.1007/s11060-016-2298-3)
Supplement: Supplementary file 1 — Supplementary material 1 (DOCX 52 KB) [file 11060_2016_2298_MOESM1_ESM.docx]

# MATERIALS AND METHODS

## Cell culture and EV isolation

Six GBM (astrocytoma grade IV) cell lines (A172, LN229, U87MG, U251, T98G and CCF-STTG1) were accessed from ATCC and cultured as previously described [11]. Cells were grown to 60% confluence, washed thrice with PBS, followed by incubation in medium with EV-depleted serum for 24 h [68]. Culture medium (~10^8^ cells) was centrifuged to pellet cells (350 x *g*, 10 min) and cellular debris (1,500 x *g*, 10 min, then 10,000 x *g*, 30 min) and then filtered (0.22-µm pore-size). The filtrate was concentrated using stirred cell ultrafiltration with a limit of 100 kDa (Millipore) and then ultracentrifuged (100,000 x *g*, 4^o^C, 16 h; Beckman Coulter Optima L-100 XP, SW32Ti). EV pellets were washed in PBS (100,000 x *g*, 4^o^C, 2 h) and stored at -80^o^C.

***Cavitron Ultrasonic Surgical Aspirator (CUSA) fluid collection and EV processing***

CUSA washings were collected during debulking surgeries of high-grade and low-grade gliomas (Sydney Local Health District HREC approval, X014-0126 & HREC/09RPAH/627) and processed within 2 h of surgery (USYD HREC approved protocol 2012/1684). Tissue fragments were strained from the CUSA fluids, formalin fixed and processed for immunohistochemistry alongside other diagnostic blocks. A specialist Neuropathologist confirmed the diagnoses and pathological changes reflective of the final diagnoses were observed in the CUSA tissue fragments. The high-grade glioma (HGG) CUSA was collected from a 59 year old female with a left frontotemporal, *IDH1wildtype* primary GBM tumour (WHO 2007 grade IV). The low-grade glioma (LGG) CUSA was collected from a 57 year old female with a left frontal, *IDH1mut* diffuse astrocytoma (WHO 2007 grade II; no 1p/19q loss detected by fluorescence *in situ* hybridization).

Cells and cellular debris were removed by centrifugation (350 x g, 10 min, RT; viability >99%) and the supernatant further centrifuged (2000 x g, 20 min, RT) followed by 0.2 µm filtration to remove and large particles. Extracellular vesicles (EV) were isolated by ultracentrifugation (100,000 x g, 4ºC, 16 h; 45 Ti, Beckman Coulter L80XP). The crude EV pellet was washed twice with sterile-filtered PBS (100,000 x *g*, 3 h, 4 ºC; 45 Ti, Beckman Coulter L80XP). The pellet was resuspended in 200 µL PBS and further purified by density gradient ultracentrifugation, using Optiprep^TM^ (60% (w/v) aqueous iodixanol from Axis-Shield PoC, Norway). Working solutions of 40% (w/v), 20% (w/v), 10% (w/v) and 5% (w/v) Optiprep^TM^ were prepared in 0.25 M sucrose/10 mM Tris, pH 7.5. From the base of a 13 mL 14x89 mm ultraclear tube (Beckman Coulter), 3 mL of 40% (w/v), then 20% (w/v) and 10% (w/v) Optiprep^TM^ working solutions were carefully layered, followed by 2.5 mL of 5% (w/v) solution. Crude EVs [300 µg protein, Qubit® fluorometric quantitation (Invitrogen, Carlsbad, CA, USA)] were diluted in 500 μL of 0.25 M/10 mM Tris, pH 7.5 and added to the top of the gradient. Gradients were ultracentrifuged (100,000 x *g*, 18 h, 4˚C, acc 1, no brake; SW41 Ti, Beckman Coulter L80XP) and 12 x 1 mL fractions of increasing density were collected. A control, blank gradient was run in parallel and each fraction was measured on an analytical balance to determine fraction density. Fractions were washed with 12 mL of PBS and ultracentrifuged (100,000 x *g*, 4 h, 4 ˚C), the pellets resuspended in 50 μL PBS and stored at -80˚C.

## Transmission electron microscopy

EVs from U87MG, LN229, CUSA HGG and LGG combined fractions 7-9 were re-suspended in dH­_2_­O, loaded onto carbon-coated, 200 mesh Cu formvar grids (ProSciTech Pty Ltd, QLD, Australia) and fixed (2.5% glutaraldehyde, 0.1 M phosphate buffer, pH7.4). Samples were negatively stained with 2% uranyl acetate for 2 min, dried for 3 h at RT and then visualised at 40,000, 80,000 and 100,000 X magnification on a Philips CM10 Biofilter TEM (FEI Company, OR, USA) equipped with an AMT camera system (Advanced Microscopy Techniques, Corp., MA, USA) at an acceleration voltage of 80-120 kV.

## Nanoparticle tracking analysis

EV size distributions and concentrations were measured by nanoparticle tracking analysis software (NTA, version 3.0) using the NanoSight LM10-HS (NanoSight Ltd, Amesbury, UK), configured with a 532 nm laser and a digital camera (CMOS Trigger Camera). Video recordings (60 s) were captured in triplicate at 25 frames/s with default minimal expected particle size, minimum track length, and blur setting, a camera level of 11 and detection threshold of 5. NTA software measured the size distribution (ranging from 10-1000 nm) and concentration of nanoparticles by simultaneously tracking Brownian motion and light scatter of individual laser-illuminated particles and calculated their diameter using statistical methods [69]. The concentration and size distribution of EVs expressing annexin A1 (ANXA1) were analysed by labelling U87MG and LN229 EVs with fluorescent antibodies. EVs (15 µg) were blocked with 0.25% (v/v) EV-depleted heat-inactivated AB serum (30 min, 4°C) and incubated with anti-ANXA1 (1 h, 4 ˚C; Abcam) followed by incubation with goat anti-rabbit Alexa Fluor® 532 conjugated secondary antibody (1 h, 4 ˚C; Thermo Scientific). ANXA1-positive EVs were measured under light scatter and fluorescent modes with a 565 nm long pass filter. NTA3.0 captured and analysed 15 s videos of the EVs at 25 frames/s in triplicate.

## EV proteome analysis

*In vitro EV analysis:* EV proteins from GBM cell lines were concentrated using chloroform-methanol precipitation, pellets dissolved in 90% formic acid (FA), concentration estimated at 280 nm using a Nanodrop (ND-1000, Thermo Scientific, USA) and aliquots dried using vacuum centrifugation. Proteomes were processed and quantified as before [11]. Peptide mixtures were resuspended in 0.05% HFBA/1% FA to a final concentration of 1 µg/µL and analysed in triplicate. EV proteomes from GBM cell lines were analyzed on an LTQ Orbitrap Velos mass spectrometer (MS; Thermo Electron, Bremen, Germany) in technical triplicate. Peptides (300 ng) were separated by nano-liquid chromatography (LC) using an Ultimate 3000 HPLC (Dionex, Amsterdam, Netherlands). Samples were first concentrated and desalted using a micro C18 precolumn (300 μm i.d.×5 mm; Dionex) with 0.05% (v/v) TFA/2% (v/v) ACN at 15 μL/min. After a 4-min wash, the pre-column (Valco 10 port valve, Dionex) was switched in line with a fritless nano C18 column (1.7 μm, 28 cm) packed with ReproSil-Pur C18-AQ stationary phase (1.9 μm, 200Å, Dr Maisch GmbH, Germany). LC mobile phase buffers were comprised of A: 0.1% (v/v) FA and B: 80% (v/v) ACN/0.1% (v/v) FA. Peptides were eluted using a linear gradient of 5%B to 42%B over 90 min at 200 nL/min. High voltage (2000 V) was applied to a low volume tee (Valco, Houston, TX, USA) and the column tip positioned ∼0.5 cm from the heated capillary (T=275^o^C) of the MS. A survey scan of 350-1750 m/z was acquired. Up to 10 of the most abundant ions (>5000 counts) with charge states ≥+2 were selected and fragmented by CID. Mass-to-charge ratios selected for MS/MS were dynamically excluded for 20 s.

*Ex vivo EV analysis:* CUSA EV peptides were analysed in technical triplicate. Peptides were re-suspended in 0.05% HFBA/1% formic acid and were separated by nanoLC using an Ultimate nanoRSLC UPLC and autosampler system (Dionex). Samples (1 μg) were concentrated and desalted onto a micro C18 precolumn (300 µm x 5 mm, Dionex) with 0.1% (v/v) trifluoroacetic acid/2% (v/v) ACN at 15 µl/min. After a 4 min wash the pre-column was switched (Valco 10 port UPLC valve, Valco, Houston, TX) into line with a fritless nano column (75µ x ~15cm) containing C18AQ media (1.9µ, 120 Å Dr Maisch, Ammerbuch-Entringen Germany). LC mobile phase buffers were comprised of A: 2% (v/v) ACN/0.1% (v/v) FA and B: 80% (v/v) ACN/0.1% (v/v) FA. Peptides were eluted using a linear gradient of 5% B to 45% B at 200 nl/min over 120 min. High voltage (2000 V) was applied to low volume Titanium union (Valco) with the column oven heated to 45°C (Sonation, Biberach, Germany) and the tip positioned ~0.5 cm from the heated capillary (T=300°C) of a QExactive Plus (Thermo Electron, Bremen, Germany) MS. A m/z 350-1750 survey scan was acquired (resolution = 70,000 at m/z 200, with an accumulation target value of 1,000,000 ions) and lockmass enabled (m/z 445.12003). Up to the 10 most abundant ions (>80,000 counts, underfill ratio 10%) with charge states > +2 and <+7 were sequentially isolated (width m/z 2.5) and fragmented by HCD (NCE = 30) with a AGC target of 100,000 ions (resolution = 17,500 at m/z 200). M/z ratios selected for MS/MS were dynamically excluded for 30 seconds. Positive ions were generated by electrospray and both MS instruments operated in data-dependent acquisition mode.

## Data analysis and bioinformatics

MS/MS data were analysed using Mascot (Matrix Science, London, UK; v2.4.0) and X! Tandem^[[1]](#footnote-1)^ (The Global Proteome Machine; vCYCLONE 2010.12.01.1) with a fragment ion mass tolerance of 0.40 Da and a parent ion tolerance of 4.0 PPM. Peak lists were searched against SwissProt databases (2014_10 for *in vitro* and 2016_03 *ex vivo* analyses), selected for *homo sapiens*, trypsin digestion, ≤2 missed cleavages, and variable modifications methionine oxidation and cysteine carbamidomethylation.

Scaffold Q+ (Proteome Software Inc., OR, USA; v4.3.2) was used to interpret proteomics data. Protein identifications were accepted if they could be established at ≥95.0% probability by the peptide prophet algorithm with Scaffold delta-mass correction and ≥2 identified peptides. Peptides used for quantification were exclusive to one protein species. Results were exported to Microsoft Excel® and SPSS® Statistics (IBM®_vr.22) for further analysis. Pearson’s r^2^ values were calculated to determine the relationship between averaged normalised precursor ion intensities of EV proteins (provided in Supplementary Table 2) and ability to produce invadopodia, expressed as %degradation relative to total cell area: U87MG (82.7%), A172 (55.8%), U251 (40.4%), T98G (29.4%), CCF-STTG1 (28.0%), and LN229 (17.5%) [11]. Significant associations were considered where r^2^>0.5 or r^2^<-0.5 at p<0.05 based on measurements from at least five cell lines. A Student t-test assuming equal variance was used to determine significant protein changes in HGG compared to LGG CUSA EV fractions. Benjamin-Hochberg (1995) FDR adjustment was applied to control for multiple testing.

EV proteins were annotated using Vesiclepedia^[[2]](#footnote-2)^ [15] and Functional Enrichment Analysis Tool^[[3]](#footnote-3)^ (FunRich; v2.1.2) [70]. Bioinformatic analyses were performed using Ingenuity® Pathway Analysis^[[4]](#footnote-4)^ (IPA) software (Ingenuity Systems, USA). Data were uploaded into the IPA environment and core analyses performed with the following amendments to default criteria:

1. Direct and indirect relationships,
2. 140 molecules per network,
3. Highly predicted or experimentally-observed confidence levels,
4. Species, human with stringent filtering,
5. Restricted to astrocytes, stem cells, nervous system tissues/primary cells, CNS cell lines or tissues.

## Western blot analysis

Protein (10 μg) from whole cell (WC) and EV proteomes from the most (U87MG) and least invasive (LN229) cell lines and EV fractions (4 μg) from the CUSA EV were analysed in triplicate with primary (PDCD6IP and ITGB1, Cell Signalling Technologies; ANXA1, Abcam), and secondary antibodies (donkey anti-rabbit IgG H&L, Abcam or goat anti-rabbit IgG H&L, Santa Cruz Biotechnology), as previously described [11].

## Gene expression levels corresponding to invasion markers in GBM tumours

Genes corresponding to the 14 significant invasion-related proteins were analysed using the Oncomine database^[[5]](#footnote-5)^ (Compendia Biosciences, MI, USA) [71]. Transcript levels were analysed in three independent cohorts (see Supplementary Tables 3 and 4 for details) [65-67]. A gene was considered significantly over/under-expressed when the mean value in tumour samples changed by >1.5 fold relative to the control mean (Student t-test; p-value<0.05). Over-expressed genes were labelled with positive integers, and under-expressed genes labelled with negative values. Pairwise t-tests were used to determine differences in *ANXA1* expression levels across the TCGA GBM transcriptional subtypes [28].

1. <http://www.thegpm.org> [↑](#footnote-ref-1)
2. <http://microvesicles.org> [↑](#footnote-ref-2)
3. <http://funrich.org> [↑](#footnote-ref-3)
4. <http://analysis.ingenuity.com> [↑](#footnote-ref-4)
5. [www.oncomine.com](http://www.oncomine.com) [↑](#footnote-ref-5)
