# Supplementary material for: Comprehensive proteome profiling of glioblastoma-derived extracellular vesicles identifies markers for more aggressive disease
Source: J Neurooncol. 2016 Oct 21;131(2):233–44. doi: 10.1007/s11060-016-2298-3 (PMC5306193; doi:10.1007/s11060-016-2298-3)
Supplement: Supplementary file 2 — Supplementary material 2 (DOCX 210 KB) [file 11060_2016_2298_MOESM2_ESM.docx]

Supplementary Table 1: EV proteins secreted by all six GBM cell lines. Evidence of detection protein in normal and cancer EVs as recorded in Vesiclepedia [14]; mRNA transcript detection by Skog *et al.* [3] and Bolukbasi *et al.* [10], and proteins previously identified in EVs secreted by U251 cells [9]; ‘#’ indicates proteins not previously reported in GBM EVs. ‘*’ Denotes top 20 exosomal proteins described by Vesiclepedia. Positive identifications in GBM and low-grade glioma CUSA EVs are indicated.

| **Acc No.** | **Protein Name** | **Gene Name** | **Vesiclepedia** | **Evidence of protein in normal EVs** | **Evidence of protein in cancer EVs** | **mRNA evidence in GBM EVs [3, 10]** | **Evidence of protein in U251 cells [9]** | **Protein identified in GBM CUSA EVs** | **Protein identified in low grade glioma CUSA EVs** |
| --- | --- | --- | --- | --- | --- | --- | --- | --- | --- |
| A2MG_HUMAN | Alpha-2-macroglobulin | A2M | VP_2 | √ | √ | X | √ | √ | √ |
| ACLY_HUMAN | ATP-citrate synthase | ACLY | VP_47 | √ | √ | √ | X | √ | √ |
| ACTN1_HUMAN | Alpha-actinin-1 | ACTN1 | VP_87 | √ | √ | √ | X | √ | X |
| ACTN4_HUMAN | Alpha-actinin-4 | ACTN4 | VP_81 | √ | √ | √ | √ | √ | √ |
| AGRIN_HUMAN | Agrin | AGRN | VP_375790 | √ | √ | √ | √ | X | X |
| SAHH_HUMAN | Adenosylhomocysteinase | AHCY | VP_191 | √ | √ | √ | X | √ | √ |
| FETUA_HUMAN | Alpha-2-HS-glycoprotein | AHSG | VP_197 | √ | √ | X | √ | √ | √ |
| ALBU_HUMAN | Serum albumin *# | ALB | VP_213 | √ | √ | X | X | √ | √ |
| ALDOA_HUMAN | Fructose-bisphosphate aldolase A | ALDOA | VP_226 | √ | √ | √ | √ | √ | √ |
| ANXA1_HUMAN | Annexin A1 | ANXA1 | VP_301 | √ | √ | √ | √ | √ | √ |
| A4_HUMAN | Amyloid beta A4 protein | APP | VP_351 | √ | √ | √ | X | √ | √ |
| ATRN_HUMAN | Attractin # | ATRN | VP_8455 | √ | √ | X | X | X | X |
| CO3_HUMAN | Complement C3 # | C3 | VP_718 | √ | √ | X | X | √ | √ |
| PYR1_HUMAN | CAD protein | CAD | VP_790 | √ | √ | √ | X | √ | X |
| CALR_HUMAN | Calreticulin | CALR | VP_811 | √ | √ | √ | X | √ | √ |
| CAND1_HUMAN | Cullin-associated NEDD8-dissociated protein 1 | CAND1 | VP_55832 | √ | √ | √ | X | √ | √ |
| TCPB_HUMAN | T-complex protein 1 subunit beta | CCT2 | VP_10576 | √ | √ | √ | X | √ | √ |
| TCPG_HUMAN | T-complex protein 1 subunit gamma | CCT3 | VP_7203 | √ | √ | √ | X | √ | √ |
| TCPD_HUMAN | T-complex protein 1 subunit delta | CCT4 | VP_10575 | √ | √ | √ | X | √ | √ |
| CD109_HUMAN | CD109 antigen | CD109 | VP_135228 | √ | √ | √ | √ | X | X |
| CSTN1_HUMAN | Calsyntenin-1 | CLSTN1 | VP_22883 | √ | √ | √ | X | X | X |
| CLH1_HUMAN | Clathrin heavy chain 1 | CLTC | VP_1213 | √ | √ | √ | √ | √ | √ |
| CLUS_HUMAN | Clusterin | CLU | VP_1191 | √ | √ | √ | X | √ | √ |
| COCA1_HUMAN | Collagen alpha-1(XII) chain | COL12A1 | VP_1303 | √ | √ | X | √ | X | X |
| CO6A1_HUMAN | Collagen alpha-1(VI) chain | COL6A1 | VP_1291 | √ | √ | √ | X | X | X |
| CO6A2_HUMAN | Collagen alpha-2(VI) chain | COL6A2 | VP_1292 | √ | √ | √ | X | X | X |
| PPGB_HUMAN | Lysosomal protective protein | CTSA | VP_5476 | √ | √ | √ | X | X | X |
| CATD_HUMAN | Cathepsin D | CTSD | VP_1509 | √ | √ | √ | X | √ | √ |
| DYHC1_HUMAN | Cytoplasmic dynein 1 heavy chain 1 | DYNC1H1 | VP_1778 | √ | √ | √ | √ | √ | √ |
| EDIL3_HUMAN | EGF-like repeat and discoidin I-like domain-containing protein 3 # | EDIL3 | VP_10085 | √ | √ | X | X | X | X |
| EF1A3_HUMAN | Putative elongation factor 1-alpha-like 3 | EEF1A1P5 | VP_158078 | √ | √ | √ | X | X | X |
| EF1G_HUMAN | Elongation factor 1-gamma | EEF1G | VP_1937 | √ | √ | √ | √ | √ | √ |
| EF2_HUMAN | Elongation factor 2 * | EEF2 | VP_1938 | √ | √ | √ | √ | √ | √ |
| EIF3C_HUMAN | Eukaryotic translation initiation factor 3 subunit C | EIF3C | VP_8663 | √ | √ | √ | X | X | X |
| EIF3L_HUMAN | Eukaryotic translation initiation factor 3 subunit L | EIF3L | VP_51386 | √ | √ | √ | X | √ | X |
| ENOA_HUMAN | Alpha-enolase * | ENO1 | VP_2023 | √ | √ | √ | √ | √ | √ |
| SYEP_HUMAN | Bifunctional glutamate/proline--tRNA ligase | EPRS | VP_2058 | √ | √ | √ | X | √ | √ |
| THRB_HUMAN | Prothrombin | F2 | VP_29251 | √ | √ | √ | X | √ | √ |
| FAS_HUMAN | Fatty acid synthase | FASN | VP_2194 | √ | √ | X | √ | √ | √ |
| FLNA_HUMAN | Filamin-A | FLNA | VP_2316 | √ | √ | √ | √ | √ | √ |
| FLNB_HUMAN | Filamin-B | FLNB | VP_2317 | √ | √ | √ | √ | √ | X |
| FINC_HUMAN | Fibronectin | FN1 | VP_2335 | √ | √ | √ | √ | √ | √ |
| GANAB_HUMAN | Neutral alpha-glucosidase AB | GANAB | VP_23193 | √ | √ | √ | X | √ | √ |
| G3P_HUMAN | Glyceraldehyde-3-phosphate dehydrogenase * | GAPDH | VP_2597 | √ | √ | √ | √ | √ | √ |
| VTDB_HUMAN | Vitamin D-binding protein # | GC | VP_2638 | √ | √ | X | X | √ | √ |
| GGH_HUMAN | Gamma-glutamyl hydrolase | GGH | VP_8836 | √ | √ | √ | X | X | X |
| GSLG1_HUMAN | Golgi apparatus protein 1 | GLG1 | VP_2734 | √ | √ | √ | X | √ | √ |
| G6PI_HUMAN | Glucose-6-phosphate isomerase | GPI | VP_2821 | √ | √ | √ | √ | √ | √ |
| GELS_HUMAN | Gelsolin | GSN | VP_2934 | √ | √ | √ | X | √ | √ |
| SYHC_HUMAN | Histidine--tRNA ligase, cytoplasmic | HARS | VP_3035 | √ | √ | √ | X | X | X |
| HBA_HUMAN | Hemoglobin subunit alpha | HBA1 | VP_3039 | √ | √ | X | √ | √ | √ |
| HEXA_HUMAN | Beta-hexosaminidase subunit alpha | HEXA | VP_3073 | √ | √ | √ | X | X | X |
| HEXB_HUMAN | Beta-hexosaminidase subunit beta | HEXB | VP_3074 | √ | √ | √ | X | X | X |
| H4_HUMAN | Histone H4 | HIST1H4A | VP_8359 | √ | √ | √ | X | √ | √ |
| HNRPC_HUMAN | Heterogeneous nuclear ribonucleoproteins C1/C2 | HNRNPC | VP_3183 | √ | √ | √ | X | X | X |
| HS90A_HUMAN | Heat shock protein HSP 90-alpha * | HSP90AA1 | VP_3320 | √ | √ | √ | √ | √ | √ |
| HS90B_HUMAN | Heat shock protein HSP 90-beta | HSP90AB1 | VP_3326 | √ | √ | √ | √ | √ | √ |
| HSP74_HUMAN | Heat shock 70 kDa protein 4 | HSPA4 | VP_3308 | √ | √ | √ | X | √ | √ |
| GRP78_HUMAN | 78 kDa glucose-regulated protein | HSPA5 | VP_3309 | √ | √ | √ | X | √ | √ |
| HSP7C_HUMAN | Heat shock cognate 71 kDa protein * | HSPA8 | VP_3312 | √ | √ | √ | √ | √ | √ |
| IQGA1_HUMAN | Ras GTPase-activating-like protein IQGAP1 | IQGAP1 | VP_8826 | √ | √ | √ | √ | √ | X |
| ITIH2_HUMAN | Inter-alpha-trypsin inhibitor heavy chain H2 # | ITIH2 | VP_3698 | √ | √ | X | X | √ | √ |
| IMB1_HUMAN | Importin subunit beta-1 | KPNB1 | VP_3837 | √ | √ | √ | X | √ | √ |
| K2C1_HUMAN | Keratin, type II cytoskeletal 1 | KRT1 | VP_3848 | √ | √ | √ | X | √ | √ |
| K1C10_HUMAN | Keratin, type I cytoskeletal 10 | KRT10 | VP_3858 | √ | √ | √ | X | √ | √ |
| K1C9_HUMAN | Keratin, type I cytoskeletal 9 # | KRT9 | VP_3857 | √ | √ | X | X | √ | √ |
| LAMA4_HUMAN | Laminin subunit alpha-4 | LAMA4 | VP_3910 | √ | GBM only | √ | X | X | X |
| LAMB1_HUMAN | Laminin subunit beta-1 # | LAMB1 | VP_3912 | √ | √ | X | X | X | X |
| LAMC1_HUMAN | Laminin subunit gamma-1 | LAMC1 | VP_3915 | √ | √ | √ | X | √ | X |
| LDHB_HUMAN | L-lactate dehydrogenase B chain | LDHB | VP_3945 | √ | √ | √ | √ | √ | √ |
| LG3BP_HUMAN | Galectin-3-binding protein | LGALS3BP | VP_3959 | √ | √ | √ | √ | √ | √ |
| LMNA_HUMAN | Prelamin-A/C | LMNA | VP_4000 | √ | √ | √ | X | X | X |
| LOXL2_HUMAN | Lysyl oxidase homolog 2 | LOXL2 | VP_4017 | √ | √ | √ | X | X | X |
| LTBP3_HUMAN | Latent-transforming growth factor beta-binding protein 3 | LTBP3 | VP_4054 | Saliva only | √ | √ | X | X | X |
| MMP2_HUMAN | 72 kDa type IV collagenase | MMP2 | VP_4313 | √ | √ | √ | X | X | X |
| MOES_HUMAN | Moesin * | MSN | VP_4478 | √ | √ | √ | √ | √ | √ |
| MYH9_HUMAN | Myosin-9 | MYH9 | VP_4627 | √ | √ | √ | √ | √ | √ |
| NUCL_HUMAN | Nucleolin | NCL | VP_4691 | √ | √ | √ | X | √ | X |
| PSA_HUMAN | Puromycin-sensitive aminopeptidase | NPEPPS | VP_9520 | √ | √ | √ | X | √ | √ |
| NPM_HUMAN | Nucleophosmin | NPM1 | VP_4869 | √ | √ | √ | X | X | X |
| P3H1_HUMAN | Prolyl 3-hydroxylase 1 | P3H1 | VP_64175 | √ | √ | √ | X | X | X |
| PDIA1_HUMAN | Protein disulfide-isomerase | P4HB | VP_5034 | √ | √ | √ | X | √ | √ |
| PUR6_HUMAN | Multifunctional protein ADE2 | PAICS | VP_10606 | √ | √ | √ | √ | √ | X |
| PDC6I_HUMAN | Programmed cell death 6-interacting protein | PDCD6IP | VP_10015 | √ | √ | √ | √ | √ | √ |
| PGK1_HUMAN | Phosphoglycerate kinase 1 | PGK1 | VP_5230 | √ | √ | √ | √ | √ | √ |
| KPYM_HUMAN | Pyruvate kinase isozymes M1/M2 * | PKM | VP_5315 | √ | √ | √ | X | √ | √ |
| PLEC_HUMAN | Plectin | PLEC | VP_5339 | √ | √ | √ | X | √ | √ |
| PLOD1_HUMAN | Procollagen-lysine,2-oxoglutarate 5-dioxygenase 1 | PLOD1 | VP_5351 | √ | √ | √ | X | √ | X |
| PLOD2_HUMAN | Procollagen-lysine,2-oxoglutarate 5-dioxygenase 2 # | PLOD2 | VP_5352 | √ | √ | X | X | X | X |
| PLOD3_HUMAN | Procollagen-lysine,2-oxoglutarate 5-dioxygenase 3 | PLOD3 | VP_8985 | √ | √ | √ | X | √ | X |
| PPIA_HUMAN | Peptidyl-prolyl cis-trans isomerase A | PPIA | VP_5478 | √ | √ | √ | √ | √ | √ |
| ANM1_HUMAN | Protein arginine N-methyltransferase 1 | PRMT1 | VP_3276 | √ | √ | √ | X | √ | X |
| SAP_HUMAN | Proactivator polypeptide | PSAP | VP_5660 | √ | √ | √ | X | X | X |
| PSA1_HUMAN | Proteasome subunit alpha type-1 | PSMA1 | VP_5682 | √ | √ | √ | X | √ | X |
| PSA3_HUMAN | Proteasome subunit alpha type-3 # | PSMA3 | VP_5684 | √ | √ | X | X | X | X |
| PSA4_HUMAN | Proteasome subunit alpha type-4 | PSMA4 | VP_5685 | √ | √ | √ | X | X | X |
| PSA5_HUMAN | Proteasome subunit alpha type-5 | PSMA5 | VP_5686 | √ | √ | √ | X | X | X |
| PSA6_HUMAN | Proteasome subunit alpha type-6 | PSMA6 | VP_5687 | √ | √ | √ | X | √ | √ |
| PSA7_HUMAN | Proteasome subunit alpha type-7 | PSMA7 | VP_5688 | √ | √ | √ | X | √ | √ |
| PSB1_HUMAN | Proteasome subunit beta type-1 | PSMB1 | VP_5689 | √ | √ | √ | X | √ | √ |
| PSB2_HUMAN | Proteasome subunit beta type-2 | PSMB2 | VP_5690 | √ | √ | √ | X | √ | X |
| PSB3_HUMAN | Proteasome subunit beta type-3 | PSMB3 | VP_5691 | √ | √ | √ | X | √ | X |
| PSB4_HUMAN | Proteasome subunit beta type-4 | PSMB4 | VP_5692 | √ | √ | √ | X | √ | X |
| PSB5_HUMAN | Proteasome subunit beta type-5 | PSMB5 | VP_5693 | √ | √ | √ | X | √ | X |
| PRS7_HUMAN | 26S protease regulatory subunit 7 | PSMC2 | VP_5701 | √ | √ | √ | X | √ | √ |
| PSD11_HUMAN | 26S proteasome non-ATPase regulatory subunit 11 | PSMD11 | VP_5717 | √ | √ | √ | X | √ | X |
| PSD13_HUMAN | 26S proteasome non-ATPase regulatory subunit 13 | PSMD13 | VP_5719 | √ | √ | √ | X | √ | X |
| PSMD2_HUMAN | 26S proteasome non-ATPase regulatory subunit 2 | PSMD2 | VP_5708 | √ | √ | √ | X | √ | √ |
| PSMD3_HUMAN | 26S proteasome non-ATPase regulatory subunit 3 | PSMD3 | VP_5709 | √ | √ | √ | X | √ | √ |
| PSMD6_HUMAN | 26S proteasome non-ATPase regulatory subunit 6 | PSMD6 | VP_9861 | √ | √ | √ | X | √ | X |
| PYGB_HUMAN | Glycogen phosphorylase, brain form | PYGB | VP_5834 | √ | √ | √ | X | √ | √ |
| QSOX1_HUMAN | Sulfhydryl oxidase 1 | QSOX1 | VP_5768 | √ | √ | √ | X | √ | X |
| RL12_HUMAN | 60S ribosomal protein L12 | RPL12 | VP_6136 | √ | √ | √ | √ | √ | √ |
| RLA2_HUMAN | 60S acidic ribosomal protein P2 | RPLP2 | VP_6181 | √ | √ | √ | X | √ | √ |
| RS18_HUMAN | 40S ribosomal protein S18 | RPS18 | VP_6222 | √ | √ | √ | √ | √ | √ |
| RS27A_HUMAN | Ubiquitin-40S ribosomal protein S27a | RPS27A | VP_6233 | √ | √ | √ | √ | √ | √ |
| RS9_HUMAN | 40S ribosomal protein S9 | RPS9 | VP_6203 | √ | √ | √ | X | √ | √ |
| RUVB1_HUMAN | RuvB-like 1 # | RUVBL1 | VP_8607 | √ | √ | X | X | √ | X |
| SEPT2_HUMAN | Septin-2 | SEPT2 | VP_4735 | √ | √ | √ | X | √ | √ |
| ANT3_HUMAN | Antithrombin-III # | SERPINC1 | VP_462 | √ | √ | X | X | √ | √ |
| GDN_HUMAN | Glia-derived nexin | SERPINE2 | VP_5270 | √ | √ | √ | X | X | X |
| SF3B3_HUMAN | Splicing factor 3B subunit 3 | SF3B3 | VP_23450 | √ | √ | √ | X | √ | X |
| 4F2_HUMAN | 4F2 cell-surface antigen heavy chain | SLC3A2 | VP_6520 | √ | √ | √ | √ | √ | √ |
| SPRC_HUMAN | Secreted protein, acidic, cysteine-rich (Osteonectin) | SPARC | VP_6678 | √ | GBM only | √ | X | √ | √ |
| SYTC_HUMAN | Threonine--tRNA ligase, cytoplasmic | TARS | VP_6897 | √ | √ | √ | X | X | X |
| TCPA_HUMAN | T-complex protein 1 subunit alpha | TCP1 | VP_6950 | √ | √ | √ | √ | √ | √ |
| TGFB1_HUMAN | Transforming growth factor beta-1 | TGFB1 | VP_7040 | √ | GBM only | √ | X | X | X |
| TSP1_HUMAN | Thrombospondin-1 | THBS1 | VP_7057 | √ | √ | √ | √ | √ | X |
| TKT_HUMAN | Transketolase | TKT | VP_7086 | √ | √ | √ | √ | √ | √ |
| TLN1_HUMAN | Talin-1 | TLN1 | VP_7094 | √ | √ | X | √ | √ | √ |
| TPIS_HUMAN | Triosephosphate isomerase | TPI1 | VP_7167 | √ | √ | √ | √ | √ | √ |
| UBA1_HUMAN | Ubiquitin-like modifier-activating enzyme 1 | UBA1 | VP_7317 | √ | √ | √ | X | √ | √ |
| UGPA_HUMAN | UTP--glucose-1-phosphate uridylyltransferase # | UGP2 | VP_7360 | √ | √ | X | X | √ | √ |
| CSPG2_HUMAN | Versican core protein | VCAN | VP_1462 | √ | √ | √ | X | √ | √ |
| VINC_HUMAN | Vinculin | VCL | VP_7414 | √ | √ | √ | X | √ | √ |
| TERA_HUMAN | Transitional endoplasmic reticulum ATPase | VCP | VP_7415 | √ | √ | √ | X | √ | √ |
| VIME_HUMAN | Vimentin | VIM | VP_7431 | √ | √ | √ | √ | √ | √ |
| VPS35_HUMAN | Vacuolar protein sorting-associated protein 35 | VPS35 | VP_55737 | √ | √ | √ | X | √ | √ |
| XPO1_HUMAN | Exportin-1 # | XPO1 | VP_7514 | √ | √ | X | X | √ | √ |
| XRCC5_HUMAN | X-ray repair cross-complementing protein 5 | XRCC5 | VP_7520 | √ | √ | √ | X | √ | √ |
| 1433B_HUMAN | 14-3-3 protein beta/alpha | YWHAB | VP_7529 | √ | √ | √ | X | √ | √ |
| 1433E_HUMAN | 14-3-3 protein epsilon | YWHAE | VP_7531 | √ | √ | √ | √ | √ | √ |
| 1433G_HUMAN | 14-3-3 protein gamma | YWHAG | VP_7532 | √ | √ | √ | X | √ | √ |
| 1433T_HUMAN | 14-3-3 protein theta | YWHAQ | VP_10971 | √ | √ | √ | √ | √ | √ |
| 1433Z_HUMAN | 14-3-3 protein zeta/delta | YWHAZ | VP_7534 | √ | √ | √ | X | √ | √ |
