# Supplementary material for: Comprehensive proteome profiling of glioblastoma-derived extracellular vesicles identifies markers for more aggressive disease
Source: J Neurooncol. 2016 Oct 21;131(2):233–44. doi: 10.1007/s11060-016-2298-3 (PMC5306193; doi:10.1007/s11060-016-2298-3)
Supplement: Supplementary file 4 — Supplementary material 4 (DOCX 48 KB) [file 11060_2016_2298_MOESM4_ESM.docx]

**Supplementary Table 3: Three independent patient cohorts used to analyze relative gene expression levels in glioma tumours.** Gene expression levels in glioma tumour specimens and control brain tissues were analysed in patient cohorts, Sun *et al.,* [18], TCGA dataset [19] and Murat *et al.,* [20]; *n* is the number of samples.

| **Cohort name** | **Platform Array** | **No. Genes analysed** | **No. Reporters** | **Control (*n*)** | **Glioblastoma (*n*)** | **Diffuse Astrocytoma (*n*)** | **Anaplastic Astrocytoma (*n*)** | **Oligodendroglioma (*n*)** |
| --- | --- | --- | --- | --- | --- | --- | --- | --- |
| Sun *et al.,* | U133 Plus 2.0 | 19,574 | 54,675 | 23 | 81 | 7 | 19 | 50 |
| TCGA dataset | U133A | 12,624 | 22,283 | 10 | 542 | - | - | - |
| Murat *et al.,* | U133 Plus 2.0 | 19,574 | 54675 | 4 | 80 | - | - | - |
