# Supplementary material for: Comprehensive proteome profiling of glioblastoma-derived extracellular vesicles identifies markers for more aggressive disease
Source: J Neurooncol. 2016 Oct 21;131(2):233–44. doi: 10.1007/s11060-016-2298-3 (PMC5306193; doi:10.1007/s11060-016-2298-3)
Supplement: Supplementary file 5 — Supplementary material 5 (DOCX 31 KB) [file 11060_2016_2298_MOESM5_ESM.docx]

**Supplementary Table 4: Reporter Information**

| **Gene Measured** | **Reporter ID** | **Nucleotide Accession** |
| --- | --- | --- |
| ANXA1 | 201012_at | NM_000700 |
| ITGB1 | 211945_s_at | BG500301 |
| ACTR3 | 213102_at | Z78330 |
| PDCD6IP | 217746_s_at | NM_013374 |
| IGF2R | 201392_s_at | BG031974 |
